# Overview of Methods to Quantify Invasiveness of Surgical Approaches in Orthopedic Surgery—A Scoping Review

**DOI:** 10.3389/fsurg.2021.771275

**Published:** 2022-01-26

**Authors:** Niels Buis, Hooman Esfandiari, Armando Hoch, Philipp Fürnstahl

**Affiliations:** Research in Orthopedic Computer Science Group (ROCS), Department of Orthopedics, University of Zurich, Zurich, Switzerland

**Keywords:** surgical approach, invasiveness, orthopedic surgery, minimally invasive, spine, hip

## Abstract

**Background:**

There is a trend toward minimally invasive and more automated procedures in orthopedic surgery. An important aspect in the further development of these techniques is the quantitative assessment of the surgical approach. The aim of this scoping review is to deliver a structured overview on the currently used methods for quantitative analysis of a surgical approaches' invasiveness in orthopedic procedures. The compiled metrics presented in the herein study can serve as the basis for digitization of surgery and advanced computational methods that focus on optimizing surgical procedures.

**Methods:**

We performed a blinded literature search in November 2020. *In-vivo* and *ex-vivo* studies that quantitatively assess the invasiveness of the surgical approach were included with a special focus on radiological methods. We excluded studies using exclusively one or multiple of the following parameters: risk of reoperation, risk of dislocation, risk of infection, risk of patient-reported nerve injury, rate of thromboembolic event, function, length of stay, blood loss, pain, operation time.

**Results:**

The final selection included 51 articles. In the included papers, approaches to 8 different anatomical structures were investigated, the majority of which examined procedures of the hip (57%) and the spine (29%). The different modalities to measure the invasiveness were categorized into three major groups “biological” (23 papers), “radiological” (25), “measured *in-situ*” (14) and their use “*in-vivo*” or “*ex-vivo*” was analyzed. Additionally, we explain the basic principles of each modality and match it to the anatomical structures it has been used on.

**Discussion:**

An ideal metric used to quantify the invasiveness of a surgical approach should be accurate, cost-effective, non-invasive, comprehensive and integratable into the clinical workflow. We find that the radiological methods best meet such criteria. However, radiological metrics can be more prone to confounders such as coexisting pathologies than *in-situ* measurements but are non-invasive and possible to perform *in-vivo*. Additionally, radiological metrics require substantial expertise and are not cost-effective. Owed to their high accuracy and low invasiveness, radiological methods are, in our opinion, the best suited for computational applications optimizing surgical procedures. The key to quantify a surgical approach's invasiveness lies in the integration of multiple metrics.

## Introduction

Surgical approaches represent the foundation on which specific interventions are built, with about 100 common surgical approaches in orthopedic surgery alone (1). A surgical approach for orthopedic procedures is defined as the technique used to reach the target bone anatomy of the intervention by resecting skin, subcutaneous fat tissue and muscle tissue. Between and within the various tissue layers lie vital structures such as nerves and blood vessels. These structures are particularly sensitive to perioperative injury as their loss of function can result in serious complications for the patient. To minimize the risk of damaging these structures, the concept of surgical planes was introduced as an important tool of modern orthopedic surgery (2). The surgical planes are explained in the order of relevance (3) (Table 1).

**Table 1:** Surgical planes.

Internervous plane	•The plane between two muscles which are innervated by different nerves. Since the muscles are separately innervated, there is no exchange of nerve fascicles between the muscles prone to potential damage.
Intermuscular plane	•The plane between two muscles innervated by the same nerve.
Intramuscular plane	•If the surgical corridor conflicts inevitably with the course of a muscle, it is favorable not to dissect the muscle completely. Instead, it is recommended to cut through the bone, as the bone with the apophysis can be reattached easily after the procedure. Alternatively, the tendinous part of the muscle should be dissected. In principle: bone before tendon before muscle.

With the goal of minimizing tissue damage, new surgical approaches are continuously being developed (4–7). Generally, these approaches can be categorized based on their invasiveness level to the following (3):

- Open approach: direct exposure of the injured parts of the target structure.
- Mini-open approach: direct exposure of the injured parts of the target structure with a small incision or multiple small incisions.
- Percutaneous approach: straight corridor from the skin to the target structure guided by medical imaging.
- Arthroscopy: camera guided technique requiring two- to three-point incisions.

Assessing the invasiveness in a more comprehensive way can be challenging given the complexity of the anatomical structures and the lack of objective and quantitative criteria based on which the surgical approach can be evaluated. The extent of the surgical incision and the quality of surgical approach can be affected by multiple factors such as: the complexity of the target anatomy and the pathology, the surgeon's level of experience and the nature of the intervention (e.g., minimally-invasive vs. open surgery). In current clinical practice either qualitative criteria (e.g., direct comparison of surgical approaches for a specific interventions) or post-operative patient outcome measures [e.g., blood loss, operation-time (8), revision-rate, infection rate (4)] are applied. However, the clinical assessment of how invasive surgical approaches are is still qualitative and lacks quantitative methods and metrics.

To the best of our knowledge, a review of quantitative metrics that could be used for assessing the invasiveness of orthopedic surgical approaches is not available in the literature. Therefore, the goal of this review article is to identify and structure all existing metrics and discuss their feasibility for integration into the clinical workflow.

## Methods

### Search Strategy

**Figure 1:**
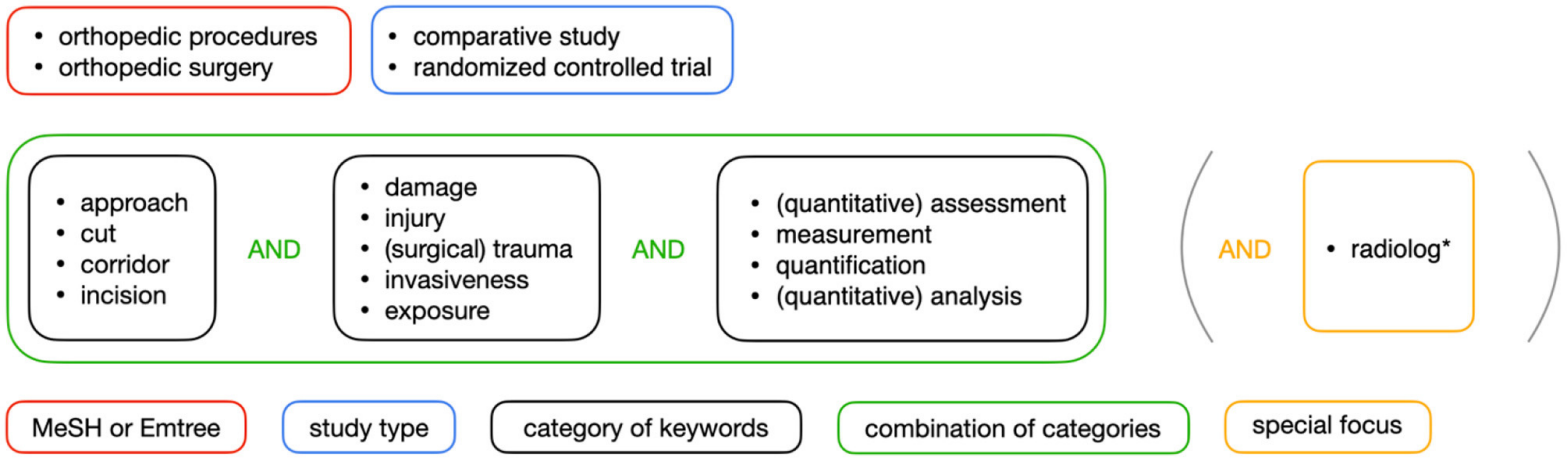
Illustration of the search strategy.

Following the guidelines published in PRISMA (Preferred Reporting Items for Systematic Reviews and Meta-Analyses), two authors independently performed a blinded literature search on Medline, Embase and Web of Science in November 2020 using the search strategy illustrated in Figure 1 (5). In order to find a broad range of articles that discuss surgical approach assessment on different conceptual levels, we used different categories of keywords that included word synonyms. Given that metrics based on radiological data are currently considered the gold standard for analyzing soft-tissue damage, we added the keyword radiolog^*^ to our search query after aggregating search results for various combinations of the three different categories of keywords.

The results of the blinded research were each cross-checked by the other author involved in the search to ensure the relevance and the adherence to the search strategy. A final list of articles was selected in a consensus meeting between the two reviewing authors.

### Study Selection and Data Extraction

Each of the articles collected was reviewed based on the abstract to identify its inclusion/exclusion according to the following criteria. We included all papers suggesting a method for quantitative evaluation of invasiveness or tissue damage caused by the surgical approach in an orthopedic procedure including spinal procedures. All publications offering only qualitative assessments were excluded. We furthermore excluded all papers using solely one or several of the following parameters for their quantitative analysis: risk of reoperation, risk of dislocation, risk of infection, risk of nerve injury, rate of thromboembolic event, functional analysis (patient-reported or with a medical score), length of stay, blood loss, number of blood transfusions, pain assessment, analgetic use rate and operation time.

There is a language bias in this publication, as we excluded all papers which were not published in or translated into English, German, Spanish, or Italian.

## Results

A total of 51 papers met our inclusion criteria and were included in the herein review.

**Figure 2:**
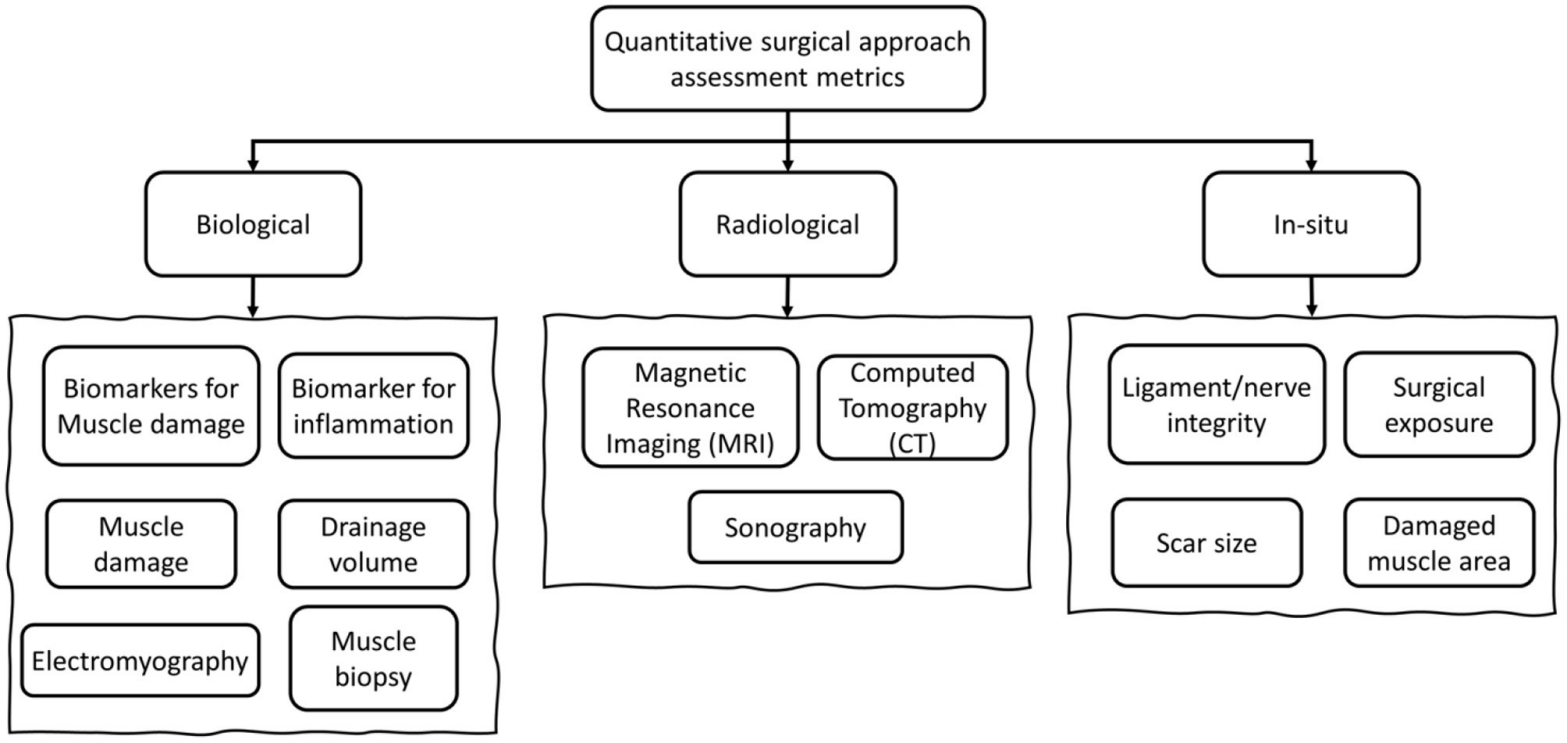
General categorization of the metrics used for surgical approach assessment.

The surgical approach assessment methods found in this review can be classified in the following three over-arching categories: biological, radiological and *in-situ* metrics (Figure 2).

A summary and categorization of the reviewed surgical approach assessment metrics is provided in Table 2.

**Table 2:** An overview of the reviewed metrics.

	**Method**	**Metric**	***in-vivo***	***ex-vivo***
Biological	Biomarkers for inflammation	CRP (6, 7, 9–12)	X	
		TNF-a (9, 11, 13)	X	
		IL-1ra (14)	X	
		IL-1ß (7, 9)	X	
		IL-6 (7, 9, 11, 13, 14)	X	
		IL-8 (13, 14)	X	
		IL-10 (13, 14)	X	
	Biomarkers for muscle damage	CK (6, 7, 9–12, 14–22)	X	
		Myoglobin (7, 11, 19, 21–23)	X	
		LDH (7, 19)	X	
		Aldolase (14)	X	
		Aspartate transaminase (7)	X	
	Electromyography	Signs of denervation (19, 24)	X	
		Recruitment of motor units (19)	X	
	Muscle biopsy	Fiber diameter (25)	X	
	Muscle strength	Dynamometer (26–28)	X	
	Drainage volume	Volume measurement (29)	X	
Radiological	MRI	Development of muscle-to-fat ratio (17, 20, 21, 23, 26, 27, 30–43)	X	
		Volume atrophy with 3D MRI reconstruction (26, 43)	X	
		Development of cross-sectional area of muscles (10, 17, 20, 28, 32, 33, 36, 37, 39, 42, 44, 45)	X	
		Integrity/quality of tendons/nerves/fascias (21, 27, 35, 41)	X	*
		Muscle edema (28, 31, 34, 35)	X	
		Bursal fluid accumulation (21, 35, 41)	X	
		Joint effusion (35)	X	
	CT	Development of cross-sectional area of muscles (32, 39)	X	
		Arc of exposure (46)	*	X
		Development of muscle-to fat-ratio (19)	X	
	Sonography	Volume of scar tissue (47)	X	
Measured *in-situ*	Surgical exposure	Volume measured with amount of saline used (48)	X	*
		Perimeter of surgical site (49)	*	X
		Measurement of exposed bone area (49)	*	X
		length and/or depth of incision (13, 16, 32, 48–51)	X	X
	Damaged muscle area (in % of total area)	Damaged cross-sectional surface area of tendons/muscles (45, 52–56)		X
	Scar size	Length of scar (18)		X
	Integrity of nerves, ligaments	*In-situ* assessment (45, 55)		X

*X: Metric has been used in the respective category*.

*: Metric could be used in the respective category, but has not been used.

### Biological Metrics

#### Biomarkers for Inflammation

These biomarkers are signaling proteins in the immune system that can be utilized for monitoring the immune activity. Such markers can be subdivided into two general categories: proinflammatory and anti-inflammatory. The biomarkers CRP (6, 7, 9–12), IL-1ß (7, 9), IL-6 (7, 9, 11, 13, 14), IL-8 (13, 14), TNF-a (9, 11, 13) act proinflammatory, whilst IL-1ra (14), IL-10 (13, 14) have an anti-inflammatory effect [as reported in Berstock et al. (14)]. After a surgical intervention, anti-inflammatory cytokines are secreted to control the inflammation in the adjacency of the incision site; therefore, the rate at which these biomarkers are released are used as an indicator for the invasiveness of the surgical approach. These biomarkers need to be measured pre- and postoperatively to achieve a point of reference for surgical approach assessment purposes.

#### Biomarkers for Muscle Damage

Intracellular enzymes are released in case of damage to the skeletal muscles (e.g., as a result of a surgical incision). Generally, these enzymes are either exclusively found in skeletal muscles cells or are notably more concentrated in skeletal muscle cells. Therefore, a systemic rise of such biomarkers in the blood circulation can be attributed to the existence of muscle damage [e.g., (15, 57)]. In order to use these biomarkers for evaluation of a surgical approach, they need to be measured pre- and postoperatively and compared, respectively.

#### Electromyography

Electromyography measures the electrical potential generated by a muscle after neurological or electrical activation. In contrast to surface electromyography, needle electromyography allows for the measurement of single motor-units by recording the generated electrical signal. Monitoring these signals before and after an incision provides a means for evaluating the surgical approach based on the damage to the underlying muscle or nerve tissue. For example, rhythmical, spontaneous contractions such as positive sharp waves and fibrillation potentials were used in Waschke et al. (19) and considered as signs of denervation, since the muscle activity functions independent of intentional activation. Alternatively, axonal injury can be used as a metric for surgical approach quality [e.g., (24)], which can be detected in the shape of polyphasic potentials and usually leads to a lower rate of motor unit recruitment. The motor unit recruitment can be measured based on the maximum voluntary contraction [as used in Waschke et al. (19) and Chomiak et al. (24)]. The more motor units are activated the more superimposition of action potentials can be seen in the electromyogram [i.e., “interference pattern”; e.g., (19)]. Muscle damage and thus a lower rate of motor unit recruitment lowers the interference pattern. Electromyography needs to be performed pre- and postoperatively to assess the postoperative development of the muscle activity and the surgical approach quality.

#### Muscle Biopsy

The development of muscle fiber diameter correlates with the muscle atrophy of the damaged muscle fibers or compensatory muscle hypertrophy of the functional muscle fibers, respectively. The assessment of the change of the muscle fiber composition due to surgical damage (e.g., ratio fast-twitch/slow-twitch) has not produced any significant results to our knowledge. Histological analysis as done in Pumberger et al. (25) needs to be performed pre- and postoperatively to measure the surgical approach quality.

#### Muscle Strength

Muscle strength can be measured using pressure sensitive sensors called dynamometers [as described in Chomiak et al. (24), Wang et al. (26), and Kim et al. (28)]. These sensors need to be pressed against with a maximum isometric contraction. It is important to position the patient in a way that the pressure measured is mainly generated by the muscle or muscle group one aims to examine. The progression of muscle strength can be utilized as a metric for assessing the surgical approach, for which the examination needs to be performed pre- and postoperatively.

#### Drainage Volume

A drainage can be placed over the operated tissue to collect the wound secretion, which itself can be used to evaluate the incision. The wound secretion is usually fluid consisting of blood and serous fluid. The volume of the collected fluid should be measured postoperatively [as reported in Chang et al. (29)] as a surgical approach metric.

### Radiological Metrics

#### MRI

Using MRI, the surgical approach of the procedure can be identified postoperatively and the damage can be assessed volumetrically (35). Therefore, magnetic resonance imaging is considered the gold standard for assessing long-term muscle damage (i.e., atrophy). The volume atrophy can be measured by manually contouring each muscle of interest using the Live-Wire software (Institute of Computing, State University of Campinas, Brazil) generating 3D reconstructions (26, 43) or by comparing the cross-sectional area of a muscle on slices of the same anatomic location on pre- and postoperative MR images [e.g., (28)]. It is important to note that the term *(fatty) atrophy* can be ambiguous, as it is sometimes used to describe the process of conversion from muscle to fat tissue [e.g., (35)] and other times to describe a reduction in volume. Therefore, we used the terms *development of muscle-to-fat ratio* (= fatty degeneration) or *volume atrophy*, respectively. An increased muscle-to-fat ratio as well as volume atrophy are the consequences of pathological processes such as immobility (58), cachexia (59), sarcopenia (60), myopathy (61), central or peripheral nervous damage (62, 63), medications (64), endocrinopathies (65). However, both forms of atrophy also occur as direct consequences of surgical trauma (66). The muscle-to-fat ratio of each muscle can further be graded according to the Goutallier classification [“grade 0 = no fat; grade 1 = few fatty streaks; grade 2 = <50% fat; grade 3 = 50 % fat, grade 4 = >50 % fat” (35)]. Furthermore, lesions of soft-tissue structures, including blood vessels (67) and nerves (68), are clearly visible in MRI volumes. Additionally, intramuscular damage can be seen as muscle edema, which is ideally depicted using a T2-weigthed sequence sensitive for fluid [e.g., (31)]. Similarly, bursal fluid [e.g., (21)] and joint effusions [e.g., (35)] can be measured in the MR image. In the included papers, certain MRI-based criteria are applied using differently weighted sequences. In all included studies, MRI was performed pre- and postoperatively.

#### CT

The development of the cross-sectional area of a muscle and the progression of the muscle-to-fat ratio can also be depicted in computed tomography scans (19, 32, 39). The muscle-to-fat ratio can be determined by distinguishing muscular from fatty tissue according to their Hounsfield units [e.g., (19)]. Analogously to MR images, the Goutallier classification can be used in CT images. The cross-sectional area of the muscle can be determined by contouring the muscle and calculating the total area using different software [Advanced Workstation (GE, USA) in Waschke et al. (19), image J (69) in Takada et al. (32) or AZE Virtual Place Raijin (Canon Inc., Tokyo) in Inoue et al. (39)]. Additionally, CT can be used to quantify the surgical exposure of different approaches. For instance in Johnson et al. (46), K-wires were placed on the outer edges of the surgically exposed capitellum using different surgical approaches. Postoperatively, the arc of exposure onto the capitellum was assessed with the help of the K-wires seen on postoperative CT volumes. Similar to MRI-based surgical approach metrics, the CT scans should be acquired pre- and postoperatively.

#### Sonography

Sonographic imaging can also be used to quantify the volume of scar tissue postoperatively (47). Superficial scar tissue is especially suitable for ultrasound imaging, because the proximity to the body surface allows the use of a high-frequency transducer, which leads to a higher resolution of the ultrasound image (70).

### *In-situ* Metrics

#### Surgical Exposure

The extent of surgical exposure can be quantified based on direct or indirect measurements of the surgical site. Belonging to the indirect category, the authors of Regev et al. (48) reported on the only case of determining the volume of the exposed surgical site based on filling the site with saline solution and measuring the volume of the solution after collecting it. In another study, anatomists photographed the exposed bone area and measured it retrospectively using an image analysis software (image J) (49). All other attempts to quantify the surgical exposure were performed with (flexible) rulers to measure: the length of incision (13, 32, 51), the depth of incision (48) or the perimeter of the surgical site (49).

#### Damaged Area

In Rossi et al. (56) the percentage of the width of a damaged muscle was assessed. Considering one dimension may be appropriate, only if the muscle/tendon is very thin at the site of measurement. However, the most common method for calculating the damaged surface area was described as follows (52): the total area of each muscle or tendon insertion was approximated by multiplying the width of the muscles' insertion by the length of insertion on the bone. The extent of the damaged (cross-sectional) area was approximated using the average length and width of torn or damaged muscle area. The authors of Van Oldenrijk et al. (45) and Lanting (55) photographed the cross-section of the muscle and imported the picture into image J (69) where the muscle area and with it the damaged surface area was retrospectively calculated.

#### Scar Size

The length of the scar is measured with a ruler in millimeters (18).

#### Integrity of Nerves and Ligaments

Postoperatively, the integrity of nerves and ligaments was evaluated *in-situ* with direct observation (45, 55).

As shown in Table 3, the majority of the studies focusing on assessing the invasiveness of different surgical approaches, have been described for hip and spine anatomy. This is owed to several factors. Firstly, THA is one of the most successful interventions in the history of orthopedics and thus very regularly performed (72). Secondly, an open and thus invasive approach is needed to place an implant at the hip joint. Thirdly, the hip is one of the largest joints in the human body – enveloped in thick layers of soft-tissue. Every approach to the hip joint causes substantial damage to the surrounding tissue in contrast to the knee which is anatomically more exposed. There are thus particularly many different possible surgical approaches to be performed. Similarly, the spine is covered by the erector spinae muscle which in addition to a complex web of nerves and blood vessels around each vertebra makes a detailed analysis of a suitable surgical corridor necessary.

**Table 3:** A breakdown of the reviewed metrics based on the underlying anatomy.

**Anatomy of interest**	**Number of papers**	**Metric**
Hip	29	- MRI: development of muscle-to-fat ratio, volume atrophy, development of cross-sectional area, joint effusion, quality of tendons/nerves/fascias, T2-signal increase (21, 23, 26, 27, 30, 32, 33, 35, 37, 38, 40, 41, 43, 71) - CT: development of cross-sectional area (32) - Surgical exposure: incision length (32) - Damaged surface area (45, 52–55) - Integrity of nerves/ligaments measured *in-situ* (45, 55) - Muscle biopsy (25) - Biomarkers for inflammation (6, 7, 9, 11, 12) - Biomarkers for muscle damage (6, 7, 9, 11, 12, 15, 18, 21–23) - Strength measurements (26, 27) - Scar size (18) - Electromyography: recruitment of motor units, signs of denervation (24)
Spine	15	- MRI: muscle-to-fat ratio development, muscle edema, development of cross-sectional area, T2-signal intensity (10, 17, 20, 28, 31, 34, 36, 39, 42, 44) - CT: muscle-to-fat ratio, development of cross-sectional area (19, 39) - Surgical exposure: volume of saline, incision length and depth (16, 48) - Biomarkers for muscle damage (10, 14, 16, 17, 19, 20, 57) - Biomarkers for inflammation (10, 14) - Strength measurements (28) - Electromyography: muscle activity, signs of denervation (19) - Drainage volume (29)
Knee	2	- Surgical exposure: perimeter of surgical site, measurement of exposed bone area, incision length (49) - Damage of muscle/tendon width (49, 56)
Elbow	1	- CT: arc of exposure (46)
Pelvis	1	- Surgical exposure: incision length (50)
Hand	1	- Sonography: volume of scar tissue (47)
Humerus	1	- Surgical exposure: incision length (51)
Femur	1	- Surgical exposure: incision length (13)

## Discussion

The herein study provides a scoping review of the available literature on the metrics used for assessing the surgical approaches used in orthopedic surgeries. By aggregating the existing metrics, we provide a categorization scheme based on the metrics' nature and the underlying anatomy. The methods found in the reviewed publications ranged widely in terms of the operational nature and therefore differed in terms of expected accuracy, expertise needed to use them, cost-effectiveness and integrability into the existing surgical workflow.

It is difficult to directly compare the expected accuracy of the different methods found in this review, as the provided data from the selected studies was heterogeneous. Additionally, almost no publication includes an explicit analysis of the used method's accuracy. However, we can assume that biological methods such as biomarkers, which measure a systemic or local reaction of the patients' body to the damage are harder to reproduce than precise *in-situ* measurements. Metrics based on biomarkers can have a large interindividual variance, rendering them difficult to use unless acquiring multiple data points (73) from the same subject. In order to use such metrics for evaluating a given surgical approach, one must determine an individual's baseline and measure the relative changes in plasma concentration to draw conclusions based on the biomarker's dynamics. Additionally, one needs to keep in mind that a rise in the biomarkers measures the systemic response, not specific to a certain locus, making this parameter prone for confounders such as a bacterial infection. In contrast, radiological methods only measure results of a local reaction to the damage and have the distinctive advantage allowing for precise quantification of the tissue shape, tissue size and of focal changes, making it possible to analyze various structures of interest individually (74).

Another key criterion a useful metric should meet is the integrability into the standard surgical/clinical workflow. We have categorized the collected metrics according to their integrability into the clinical workflow (Table 4).

**Table 4:** Integrability of the surgical approach assessment metrics.

Already integrated in clinical workflow	•Blood sampling for biomarkers •CT •Drainage volume
Easy to integrate into clinical workflow	•Surgical exposure •Muscle strength •MRI •Scar size •Sonography
Unsuitable for clinical workflow	•*In-situ* measurement of damaged area •Integrity of nerves, ligaments •Muscle biopsy •Electromyography

Preoperative radiography and/or preoperative CT are acquired routinely for the planning of orthopedic procedures (75). For certain procedures such as bone tumorectomy (76) or the treatment of scoliosis (77) a preoperative MRI is indicated. Nonetheless, MRI is rather a diagnostic imaging modality than a routinely used tool in preoperative planning (78). Therefore, it should be highlighted that the integration into the clinical workflow can be easier for metrics that are based on CT imaging compared to the metrics that are based on MRI. On the other hand, MRI is a non-invasive technique which, is still integrated more easily than invasive techniques such as biopsies that cannot be used in clinical routine for surgical approach quantification. In general, all types of biomarker-based metrics are effortlessly adoptable into the clinical workflow as blood sampling is part of the preoperative routine and is often performed postoperatively as well. Almost as smoothly integratable are the postoperative scar size measurement and the measurement of the exposed volume using saline solutions as they require modest extra time. Other methods such as electromyography are less suitable for clinical adoption since they require additional hardware and special knowledge to adequately measure the surgical approach quality.

In terms of cost-effectiveness, biomarkers are in general cost-effective in comparison to the gold standard method using radiological imaging such as MRI (79, 80). An MRI needs to be made pre- and postoperatively to objectively assess the development of the soft tissue after a procedure.

This gives a better foundation for interindividual comparison. Clearly, the choice of metric needs to be evaluated based on individual situations.

Attempts have been made to compare the invasiveness of different procedures objectively with a score (81, 82). These scores are useful to obtain a general idea of the level of invasiveness. However, we consider them unfit to distinguish between different surgical approaches, as it is more the operation site and the nature of the procedure which influence the score. Thus, we believe that in order to evaluate and reliably compare different surgical approaches, a more extensive and broader selection of metrics is required.

In the recent years, there has been an increasing interest for designing artificial intelligence models that contribute toward optimizing currently used surgical protocols [e.g., (83)]. Therefore, we expect that in the future, the demand for autonomous or semi-autonomous surgical assistance in orthopedic interventions such as: computer assisted surgical navigation [e.g., (84)], surgical robotics [e.g., (85)] and medical artificial intelligence [e.g., (86, 87)] will increase. Many of the artificial intelligence methods are based on deep learning algorithms, which generally rely on a large training dataset and learn to replicate similar patterns when confronted by new data. In order to facilitate the clinical adoption of such methods, robust metrics for assessing the current or the intended surgical approach are required. In our opinion, metrics that are based on radiological and *in-situ* measurements are more suited for such models given that in principle they rely on direct observations of the underlying anatomy, while the biological metrics on the other hand are complex physiological reactions and offer data points that depend on an individual baseline with large interindividual variance. Preoperative estimation of the extent of damage based on a combination of radiological, biological or *in-situ* measured data from comparable patient groups may be possible in the future. However, the use of patient- and anatomy-specific data may allow personalized decision making [i.e., choosing the adequate surgical approach for the patient's anatomy (88)]. For this purpose, radiological metrics should be preferred as they can be acquired non-invasively. Not only the correct choice of approach, but also the necessary surgical expertise contributes toward improving patient care. Therefore, training software such as Touch Surgery (Medtronic, Ireland) or Incision (Incision Group B.V., Netherlands) yield notable potential for the optimization of surgical approaches (89).

## Limitations

This review cannot contribute toward assessing the accuracy of the listed metrics. For that purpose, a comparison between the clinical outcome and the data produced by a certain metric would need to be made. Additionally, further research is needed to evaluate which metric is ideal for the approach assessment of different anatomical structures. Finally, many regularly used metrics such as blood loss or operation time were not discussed in this review.

## Conclusion

Ideally, the metrics used to quantify the invasiveness of a surgical approach should be accurate, cost-effective, non-invasive, comprehensive and integratable into the clinical workflow. After detailed consideration of all novel metrics used in the literature, we find that the radiological methods (development of muscle-to-fat ratio, volume atrophy, development of cross-sectional area, joint effusion, muscle edema, T2-signal increase and quality or integrity of tendons/nerves/fascia) best meet those criteria. However, such metrics can be more prone to confounders such as coexisting pathologies than *in-situ* measurements but are non-invasive and possible to perform *in-vivo*. Additionally, radiological metrics do require a lot of expertise and are not the most cost-effective.

We can thus conclude that there is no metric that delivers a complete and objective quantitative analysis of the surgical approach. The key lies in the integration of multiple metrics, allowing an extensive analysis of the invasiveness of a surgical approach. Moreover, further research needs to be conducted for assessing the quality of the proposed methods by comparing their correlation with the functional outcomes of the procedures.

## Author Contributions

PF, HE, and NB contributed to the ideation of the review. NB and HE performed the literature research and made the selection of papers. NB extracted and structured the relevant contents of each paper of the selection and wrote the first draft of the manuscript. HE wrote sections of the manuscript. All authors contributed to manuscript revision, read, and approved the submitted version.

## Funding

HE salary was partially funded by the SURGENT project which was financed by a University Medicine Zurich, University of Zurich seed funding.

## Conflict of Interest

The authors declare that the research was conducted in the absence of any commercial or financial relationships that could be construed as a potential conflict of interest.

## Publisher's Note

All claims expressed in this article are solely those of the authors and do not necessarily represent those of their affiliated organizations, or those of the publisher, the editors and the reviewers. Any product that may be evaluated in this article, or claim that may be made by its manufacturer, is not guaranteed or endorsed by the publisher.
